# Bhutanese science teachers’ perceptions of the nature of science: a cross-sectional study

**DOI:** 10.1186/s43031-021-00044-9

**Published:** 2022-02-07

**Authors:** Karma Dorji, Sherab Jatsho, Pem Choden, Pema Tshering

**Affiliations:** 1 Curriculum Development Centre, Royal Education Council, Shari, Post Box No 1243, Paro, 12001 Bhutan; 2 Department of Science, Yangchen Gatshel Central School, Thimphu, 11001 Bhutan; 3 Department of Science, Damphu Central School, Damphu, 36001 Bhutan; 4 Department of Science, Rangjung Central School, Tashigang, 42005 Bhutan

**Keywords:** Bhutanese science education, Bhutanese science teachers, Nature of science, Cross-sectional study

## Abstract

This study investigated Bhutanese science teachers’ conceptions of the nature of science (NOS). The study recruited 225 Bhutanese science teachers based on convenient and snowball sampling techniques. The data was collected using the Myths of Science Questionnaire (MOSQ). The MOSQ was designed on Google Forms and administered through the online survey mode. The data was analysed using descriptive statistics in terms of the measure of frequency supported by science teachers’ open-ended written responses, Independent Sample *t*-test, and One-way Analysis of Variance (ANOVA). Findings from descriptive statistics showed that Bhutanese science teachers considerably lacked clear understanding of the NOS in terms of scientific knowledge, scientific method, scientists’ work, and scientific enterprise. The Independent Sample *t*-test showed that there was no statistically significant difference between Bhutanese male and female science teachers’ perceptions of the NOS with *p* > .05. The One-way ANOVA test revealed statistically significant differences amongst Bhutanese science teachers’ perceptions of the NOS based on their academic qualifications with *p <* .05. The Tukey Post-hoc test, however, revealed that Bhutanese science teachers’ perceptions of the NOS based on academic qualifications was significant only between teachers with postgraduate diploma and doctor of philosophy.

## Introduction

Science is virtually involved in every sphere of our life. It is common in almost every part of political, social, and economic tools ranging from the marvels of technology to the impacts of principles and philosophical assumptions (Khishfe, 2012). Although science is prominent in its influence, it is quite common that some science professionals hold a naive conception of how science works or the characteristics of science in itself (Bell et al., 2011; Bell et al., 2012). By far, such a naive understanding of the nature of science (NOS) is commonly considered harmful, especially when citizens are required to make informed decisions, evaluate policy matters, and make a judgment over scientific pieces of evidence. At the centre, many irrational plans and decisions, either wholly or in part, are attributed decidedly as the consequence of a lack of clear understanding of the NOS (Allchin et al., 2014; Lederman, Antink, and Bartos, 2014). The NOS, therefore, is one of the domains of science that everyone needs to understand (Deng et al., 2011; National Science Teaching Association, 2013; Oslon, 2018).

Considering the need to build a clear understanding, the NOS today is recognised as one of the cornerstones of scientific literacy (Dagher & Erduran, 2016; Kartal et al., 2018; National Research Council, 2012; National Research Council, 2013). Perhaps, the development of a matured understanding of the NOS is the perennial aim of science education around the world (Chaisri & Thathong, 2014; Nuangchalerm, 2010). The scientific community, therefore, believes that there must be a science education that places a strong emphasis on the understanding of science concepts, principles and theories, and the processes of science in itself (Torres & Vasconcelos, 2015). Moreover, science professionals and science educators commonly agree that there must be a science education that helps to understand the shared relationships of science, technology, and society; and the understanding of the NOS itself (Torres et al., 2015).

Science education today recognises the importance of featuring the NOS in science curricula (Kartal et al., 2018; Prachagool & Nuangchalerm, 2019). The science curricula, though vary across the world, aspirations to help students understand the aspects of the NOS is one thing that remains common across the curricula (Irzik & Nola, 2014; Kaya & Erduran, 2016). Students’ understanding of the NOS, thus, is largely emphasised as an important curricular objective of science curricula worldwide (Lederman, Bartos, and Lederman, 2014).

## Review of literature

### The NOS

The NOS is largely complex given its multifaceted endeavour. As there is no fixed definition of the NOS, philosophers, historians, and science educators maintain different definitions or meanings of the NOS. Therefore, the NOS is largely difficult for experts to define as much as it is difficult for students to learn (Sumranwanich & Yuenyong, 2014). At the core, however, scientists and science educators agree with the NOS from certain parameters. That is, the NOS is understood as the epistemology of science, science as a way of knowing, or the values and assumptions that form scientific knowledge (Lederman, 2007; Lederman et al., 2013). Moreover, the NOS is also commonly referred to as a characteristic of scientific knowledge. By this point, the NOS means largely but explicitly the way the scientific knowledge is constructed (Lederman, Antink, and Bartos, 2014; Lederman and Lederman, 2014). In their stand, McComas et al. (1998) describe the NOS as:

The nature of science is a fertile hybrid arena, which blends aspects of various social studies of science including the history, sociology, and philosophy of science combined with research from the cognitive sciences such as psychology into a rich description of what science is, how it works, how scientists operate as a social group and how society itself both directs and reacts to scientific endeavours. (p. 4).

To a large extent, the NOS is commonly understood in terms of four basic constructs: scientific knowledge, scientific method, scientists’ work, and scientific enterprise (Buaraphan, 2010). These constructs include some of the aspects of the NOS: (a) scientific knowledge is tentative; (b) scientific knowledge relies heavily upon, but not entirely, on observation, experimental evidence, rational arguments, and scepticism; (c) scientific knowledge is a human construct; (d) partly the product of inference, imagination, and creativity; (e) there is no universal step-by-step scientific method; (f) laws and theories serve different roles in science; (g) observations are theory-laden; scientists are creative; (h) science and technology impact on each other; and (i) scientific ideas are affected by their social and historical milieu (Abd-El-Khalick 2012; Mesci & Schwartz, 2017).

### Science teachers’ view of the NOS

Given that the NOS is one of the cornerstones of science education, science teachers must have a better understanding of the NOS. Science teachers’ conceptions of the NOS have a direct impact on the quality of their daily classroom instruction (Lederman and Lederman, 2019a, Lederman and Lederman, 2019b). Therefore, science teachers must have an adequate understanding of what they attempt to teach about the NOS (Lederman, 1992). To a greater extent, science teachers cannot possibly teach what they do not understand about the NOS (Capps et al., 2012; Clough, 2018; Herman & Clough, 2016). As such, without informed views of the NOS, science teachers would possibly fail to project the NOS in their classroom teaching (Capps & Crawford, 2013).

With support from scientists and the scientific community, there is a general belief that teachers have an adequate understanding of the NOS. However, it seems that there is little progress made in terms of achieving educational goals, especially about the NOS. Therefore, a growing body of research reports that views of the NOS, both from teachers and students, are often dissatisfying or uncertain (Lederman, 2007; Lederman and Lederman, 2014, 2019a); mixed, fluid and incoherent (Buaraphan (2009); or shallow and incongruent to the view of scientists and the scientific community (Aslan & Tasar, 2013; Ibrahim et al., 2009; McDonald, 2010).

As per Dogan and Abd-El-Khalick (2008), many science teachers often come with the notion that when a hypothesis is proven correct, it becomes a theory and when a theory is proven correct, it becomes a law. In conjunction with this view, many science teachers also misappropriate scientific theories as less secure compared to laws (Akerson et al., 2006). Given this, science teachers oftentimes believe in the diehard myth that scientific theories become laws only after being proven true many times by enough evidence or different people, and have been around for a long time (Akerson et al., 2006). In studies conducted by Aslan and Taser (2013) and Dogan and Abd-El-Khalick (2008), many Turkish science teachers expressed naive conceptions regarding the hierarchical relationship amongst hypotheses, theories, and laws.

Many science teachers also assume that scientific knowledge becomes more stable with the availability or the accumulation of evidence. This misattributed notion of science teachers was found out by Ma (2009) in the study conducted to examine Chinese science teachers’ conceptions of the NOS. Besides, science teachers also commonly believe and carry on with an idea that scientific knowledge is both durable and reliable (Jain et al., 2018). With such a type of understanding, science teachers often regard science as static or have a static status.

In the other aspect, science teachers easily misunderstand scientific models as copies of realities (Guerra-Ramos et al., 2010). Moreover, they also often take the scientific method as a fixed step-by-step method or believe in the recipe-like notion of doing science (Mesci & Schwartz, 2017). With such misinformation, science teachers, oftentimes, take science as a lifeless, rational, and orderly activity (Abd-El-Khalick, 2012). Many science teachers also viewed scientists as free of biases and prejudices (Prachagool & Nuangchalerm, 2019); and creativity and imagination are not the endeavours of scientists (Akerson & Donnelly, 2008). With such a view, science teachers often miss an understanding that science in itself is a direct result of human endavour, creativity and imagination. Science teachers also misinterpret science and technology as the same facet or same entity (Bell et al., 2016). Therefore, with such misunderstanding in place, there is a renewed focus from science education reform initiatives in improving science teachers’ conceptual understanding of the NOS (Wahbeh & Abd-El-Khalick, 2014).

### The NOS based on gender and education level

#### Gender and the NOS

The current research on the influence of gender on teachers’ NOS conception is quite limited. A few studies report that there is no significant relationship between science teachers’ gender and their conceptions of the NOS (Saif, 2016; Oluwatayo, 2011; Taale, 2014; Yaman & Nugoglu, 2010). For instance, the descriptive survey carried out by Adedoyin and Bello (2017) and Ajaja (2012) Nigeria revealed an equal proportion of male and female secondary science teachers with a traditionalist and/or constructivist view of the NOS. In their study of the 201 Turkish science teachers, Yaman and Nugoglu (2010) also found out that the conceptions of the NOS held by male science teachers are statistically not significant from the conceptions of the NOS held by female science teachers.

In most parts of science education literature, the lack of correlation between science teachers’ gender and their NOS conceptions is attributed mostly to the educational setting placed around them (Oluwatayo, 2011; Taale, 2014). That is if teachers receive training through the same curricula, same instructors, or similar processes, their NOS conceptions invariably remain the same or similar irrespective of their gender (Adedoyin & Bello, 2017; Saif, 2016). As per Ajaja (2012), both male and female science teachers would have the highest instances to see the NOS from a similar lens if they handle the same curriculum in their daily classroom teaching, including textbooks. On the other hand, Dogan and Abd-El-Khalick (2008) espouse reasons from different and bigger lines of thought. For them, teachers exposed to the same social-cultural aspects would only ascribe the NOS from a similar viewpoint.

#### Academic qualifications and the NOS

The influence of science teachers’ academic qualifications or educational levels upon the NOS conceptions is quite straight to the point. Contrary to the common belief, there is no significant relationship between science teachers’ academic qualifications and their conceptions of the NOS (Carey & Stauss, 1970; Lederman, 1992; Mellado, 1997). For example, in the study conducted by Dogan and Abd-El-Khalick (2008), Grade 10 Turkish science teachers holding master’s degrees expressed more naive views than those holding bachelor’s degrees, while those holding doctor of philosophy had the most misinformed conceptions. A similar trend was also observed by Bruckermann et al. (2018) in their assessment of German pre-service biology teachers. On the contrary, Ajaja (2012) in his survey found Nigerian science teachers with Bachelor of Science (BSc.) with the most informed views of the NOS followed by those with BSc. in Education and the Nigerian Certificate of Education (NCE).

All in all, there is no guarantee that teachers with better academic qualifications have a better understanding of the NOS. As inferred by Bruckermann et al. (2018), teachers ‘view of the NOS would rather be markedly influenced by the amount of NOS learning opportunities they receive during their teacher preparatory programme. For them, BSc. degree teachers with more exposure to the NOS learning opportunities would invariably have a better understanding of the NOS than those with a master’s degree or doctor of philosophy. As per Ajaja (2012), the NOS conceptions held by science teachers is more connected with the number of science concepts and the methods of doing science received during teacher preparatory programmes.

### The NOS in Bhutan

The concept of the NOS is relatively new to Bhutan. As opposed to the report of Das et al. (2017), the need for the NOS is explicitly captured in the Bhutanese science curriculum framework. According to the Royal Education Council (REC) (2012), the larger intention of Bhutanese science education is to “develop in the learner the notion of scientific temper …. give learners a strong foundation in science …. develop citizens that can make informed decisions” (p. 8). In this connection, one of the goals of Bhutanese science education is to “enable the learners to appreciate that while science can answer most questions, there are also questions which cannot answer” (Royal Education Council, 2012, p. 16). More so, the rationale of science practical works conducted in Bhutanese science classrooms demands science teachers to help learners understand the nature of scientific knowledge and how science works (Royal Education Council, 2016). The Bhutanese science curriculum framework acknowledges that the quality of understanding of the nature of scientific knowledge and how science works by students will largely depend on the level of Bhutanese science teachers’ understanding of the NOS.

Research on the NOS, especially in the Bhutanese context, is rare. The study carried out by Wangdi et al. (2019) is the only study that reports about the Bhutanese science teachers’ perceptions of the NOS. Using a survey questionnaire, they have collected data from 78 Bhutanese science teachers gathered in one of the engineering colleges in Bhutan. Statistically, they found that Bhutanese science teachers have a low level of understanding of the NOS in the area of scientific knowledge, scientific method, scientists’ work, and scientific enterprise. Therefore, to date, there is virtually no study that provides a qualitative report on Bhutanese science teachers’ understanding of the NOS. Despite their findings, Wangdi et al. (2019) also suggest carrying out a further inquiry to understand both the level and a qualitative view of the NOS held by Bhutanese science teachers involving a larger sample size. Thus, this study was carried out to ascertain both the level and the qualitative understanding of the NOS held by Bhutanese science teachers. The research had the following questions:

1. What are Bhutanese science teachers’ perceptions of the NOS in terms of scientific knowledge, scientific method, scientists’ work, and scientific enterprise?
2. Is there a significant difference in the Bhutanese science teachers’ perceptions of the NOS based on gender and academic qualification?

Findings from this study were expected to inform Bhutanese policymakers, education officials, and curriculum developers regarding the level and the nature of Bhutanese science teachers’ perceptions of the NOS. As a result, it was expected that Bhutanese policymakers, education officials, and curriculum developers make an informed decision on policy-related and curricular matters; and provide a basis to design intervention programmes related to the NOS. This study was also expected to provide views on the Bhutanese science teachers’ perceptions of the NOS to international science education forums. Besides, the study also sought science education forums from other parts of the world to adapt and compare Bhutanese science teachers’ perceptions of the NOS in their contexts.

## Materials and methods

### Research design

This study used the cross-sectional study to document Bhutanese science teachers’ perceptions of the NOS (Sedgwick, 2014). It was the non-experimental study design that captured nationwide snapshot views of the NOS held by Bhutanese science teachers. The study was driven by the premise of the positivist approach that attempted to fit teachers’ conceptions of the NOS in terms of scientific knowledge, scientific method, scientists’ work, and scientific enterprise. The research employed both descriptive and inferential statistical inquiry methods to understand Bhutanese science teachers’ perceptions of the NOS.

### Sample

This study administered the MOSQ to more than 250 Bhutanese science teachers. Only 225 Bhutanese science teachers, however, responded to the MOSQ. As shown in Table 1, the study recruited science teachers from five major regions of Bhutan. The maximum number of Bhutanese science teachers came from western region followed by the southern and eastern regions. Science teachers were drawn into the study based on convenient and snowball sampling techniques. Schools in Bhutan remained closed for a few months due to the COVID-19 pandemic during the study. This made it difficult to contact or locate the majority of science teachers. Therefore, the researchers recruited science teachers who remained easy to contact or locate. Besides, the study also recruited science teachers based on the assistance of science teachers who were already drawn in as the study sample. The researchers also sought assistance from school principals and teachers majoring in other subjects in identifying potential science teachers.

**Table 1:** The Number of Participating Science Teachers Based on Region

Region	Number of Science Teacher
West	85
East	58
Central	9
North	7
South	66

The sample of the study was composed of both male (*n* = 157) and female (*n* = 68) Bhutanese science teachers. They were full-time science teachers who had formally graduated from colleges of education. They ranged in age from 24 to 47 with a wide range of teaching experiences. The vast majority (*n* = 157) of science teachers had more than three years of teaching experience. Many of them had their pre-service training from colleges of education in either a bachelor’s degree in education (B. Ed) or a post-graduate diploma in education (PGDE). A large number (*n* = 120) of them had three or four years of B. Ed pre-service training courses, whereas the rest had undergone one-year PGDE pre-service training courses. They (*n* = 79) also had master’s degrees either in education (M. Ed) or in specific science disciplines (MSc) in addition to their pre-service training. Interestingly, nine Bhutanese science teachers had a doctorate of philosophy (PhD).

The majority (*n* = 84) of science teachers were biology teachers followed by physics teachers (*n* = 52), and chemistry teachers (*n* = 42). There were science teachers from general science (*n* = 38) and environmental science (*n* = 9) teaching backgrounds. A large number (*n* = 123) of them had the experience of teaching Grade 7 to 10, while slightly more than one-quarter (*n* = 66) of them had the experience of teaching Grade 11 and 12. While thirty-four of them had the experience of teaching pre-primary (PP) to Grade 6, the rest had the experience of teaching more than one level of grade range. That is thirteen of them had the experience of teaching PP to Grade 6 and Grade 7 to 8, whereas, others had the experience of teaching Grade 9 to 12.

### Instrument

To understand Bhutanese science teachers’ conceptions of the NOS, this study adopted the Myths of Science Questionnaire (MOSQ) designed by Buaraphan (2009). The MOSQ contained 14 Likert-type close-ended items spread in the four constructs: scientific knowledge, scientific method, scientists’ work, and the scientific enterprise as shown in Table 2. The MOSQ item 1, 2, 3, 4, 8, and 9 belonged to scientific knowledge constructs, whereas items 5, 6, and 7 belonged to scientific method constructs. Item 10 and 11 belonged to scientists’ work, while items 12, 13, and 14 belonged to scientific enterprise constructs. Responses to the MOSQ items entailed the respondents to select one of the levels from three choices: *agree, uncertain*, or *disagree*, which best suits their perception of the item statements. Besides, the MOSQ also entailed respondents to provide subjective explanations. Typically, subjective responses entailed respondents to make their point of justification for the choice made from three levels of choices against each Likert-type item.

**Table 2:** MOSQ items as per the Constructs

Construct	Item
Scientific knowledge	1, 2, 3, 4, 8, & 9
Scientific method	5, 6, & 7
Scientists’ work	10 & 11
Scientific enterprise	12, 13, & 14

The MOSQ was validated first by the panel of five science educators. The MOSQ items were validated in terms of their congruence with the constructs of the NOS; and their comprehensibility and appropriateness to respondents. Based on comments from experts, the MOSQ items were revised and piloted with 21 pre-service and 11 in-service science teachers in one of the central regions of Thailand. The piloting of the MOSQ items was carried out to determine whether science teachers understood the test items and the amount of time they would spend completing the MOSQ. Based on uncertainties found during the pilot test, the MOSQ items were revised further.

The study carried out by Sarkar and Gomes (2010) to examine Bangladeshi science teachers’ perceptions of the NOS established the test reliability of the MOSQ items. The study found out the MOSQ items with relatively high internal consistency. The MOSQ items had Cronbach’s alpha reliability coefficient of 0.79.

### Data collection

The data was collected in October 2020 spanning from the mid of the month to the end of October. Data was collected through the online survey mode via e-mail correspondence. For this, the MOSQ adopted from Buaraphan (2009) was designed on Google Forms. The researchers collected e-mail addresses of science teachers from school principals, vice-principals, or science teachers themselves. Concurrently, e-mail addresses were also gathered directly by contacting non-science teachers familiar to the researchers. In certain cases, the researchers also sought assistance from science teachers and non-science teachers in sharing the MOSQ with science teachers who were familiar with them. After collecting the list of e-mail addresses, the MOSQ designed on Google Forms was shared with more than 250 science teachers. The data collection procedure followed the due process of ethical standards. The science teachers were informed about the rationale of the study through the MOSQ. The study sought informed consent from each science teacher. Moreover, science teachers were also informed about how their participation in the study will remain confidential. The “accepting responses” button on Google Forms was disabled by the end of October 2020 when the researchers did not see any further additional responses. Subsequently, data was downloaded from Google Forms in Microsoft Excel Sheet for analysis.

### Data analysis

The data collected through the MOSQ was analysed using both descriptive and inferential statistical methods. The descriptive statistical methods were carried out to determine Bhutanese science teachers’ perceptions of the NOS in terms of the measure of frequency using Microsoft Excel 2013. The frequency of response to each MOSQ item was grouped into three categories−*agree, uncertain,* and *disagree*. Subsequently, the frequency of response to each category (*agree, uncertain,* and *disagree*) was computed in terms of percentage. Further, the frequency of response to the *agree* category was labelled as uninformed conceptions of the NOS (informed view for item number 8), while the frequency of response to the *disagree* and *uncertain* category were described as informed and uncertain conceptions respectively (uninformed and uncertain view for item number 8) (Buaraphan, 2009).

Concurrently, written responses were extracted to generate codes based on the deductive approach of content analysis. The MOSQ items formed the theory or pre-determined category for the coding process. As such, each code that appeared important, interesting, or related to the MOSQ items were grouped into one category. Written responses or codes, thus, formed the point of justification for each category of response. Direct quotes from teachers’ responses were also used to support claims wherever required.

In inferential statistical methods, responses to the MOSQ items were scored ranging from 0 to 2. Response to the *agree* category was scored 0, while the response to the *uncertain* and *disagree* categories were scored 1 and 2 respectively. However, for item number 8, the response to the *agree* category was scored 2, while the response to the *uncertain* and *disagree* categories were scored 1 and 0 respectively. Using the grand mean score of each respondent, the Independent Samples *t-*test and One-way Analysis of Variance (ANOVA) were carried out using Statistical Package for Social Sciences (SPSS) version 20. The Independent Samples *t-*test compared Bhutanese male and female science teachers’ perceptions of the NOS, while One-way ANOVA compared Bhutanese science teachers’ perceptions of the NOS based on their academic qualifications. Compared means from both the Independent Samples *t-*test and One-way ANOVA were tested at a .05 level of significance (95% confidence interval).

## Results

Bhutanese science teachers’ perceptions of the NOS are reported broadly in terms of the measure of frequency and compared means as:

### Perceptions based on the measure of frequency

Bhutanese science teachers’ perceptions of the NOS in terms of the measure of frequency are reported in four constructs as:

#### Perceptions of the NOS: scientific knowledge

Bhutanese science teachers’ perceptions of the NOS concerning scientific knowledge are illustrated in Table 3.

**Table 3:** Perceptions of the NOS: Scientific Knowledge (*N* = 225)

Items	Response (%)		
	Agree	Uncertain	Disagree
Item 1: Hypotheses are developed to become theories only	38.8	21.0	40.2
Item 2: Scientific theories are less secure than laws	39.6	24.4	36.0
Item 3: Scientific theories can be developed to become laws	77.7	12. 5	9.8
Item 4: Scientific knowledge cannot be changed	15.1	12.1	72.8
Item 8: Accumulation of evidence makes scientific knowledge more stable	96	1.8	2.2
Item 9: A scientific model (e.g., the atomic model) expresses a copy of reality	71.6	18.2	10.2

Many (38.8%) Bhutanese science teachers expressed the uninformed view that “hypotheses are developed to become theories only”. With little or no understanding, they maintained that hypotheses, in any event, are always developed to become theories at one point in time. As they appeared certain of their view, they justified that this happens often, if not, on all occasions in light of mounting evidence; advancement in reasons and thinking; or professional scrutiny of ideas and concepts by scientists. Nearly two-fifths (39.6%) of the Bhutanese science teachers also believed that “scientific theories are less secure than laws”. Surprisingly, a vast majority (77.7%) of them also expressed uninformed views of the hierarchical relationship shared by scientific theories and laws. According to them, scientific theories, usually, if not, routinely become laws with the availability of evidence or advancement in theories and understanding. With such a misinformed view, their justification was such that scientists develop scientific theories first and develop laws either in light of shreds of evidence or when arguments are backed up by logic and reasons. Given below is an excerpt from one of the responses of a Bhutanese science teacher:

*“Hypotheses are some forms of educated guesses purported to make theories and then laws. With more pieces of evidence, hypotheses become theories; and with additional shreds of evidence, theories further become laws. Laws are stronger than theories”.*

Significantly, the overwhelming majority (72.8%) of the Bhutanese science teachers held informed views about the tentativeness of scientific knowledge. As they were confident of their claim, they thought that scientific knowledge, though durable and reliable, is never absolute and static. A body of scientific knowledge, such as theories, laws, or principles, as per their justifications, do not remain fixed or achieve the supreme status. As such, many of them instead hinted that all scientific knowledge, such as theories, laws, or facts are certain to get revised over time with the advent of new, plausible, or intelligible evidence. At the same time, others believed that both scientific knowledge and the course of scientific progression, as being socially embedded, are shaped steadily by social values. The excerpt below is one of the responses of a Bhutanese science teacher:

*“Science is neither static nor stable. With a discovery, old knowledge, such as theories, concepts, principles, or laws become unstable or get disapproved. For example, the discovery of atomic structure dates back to John Dalton’s atomic model. His theory was replaced by J. J Thomson’s atomic model while J. J Thomson’s atomic model was disapproved later by Bohr’s atomic model*”.

Almost all (96%) the Bhutanese science teachers believed in the cumulative nature of scientific knowledge. For this, they expressed two schools of thoughts. A large group of them opined that scientific knowledge, as it does in any occasion, often becomes stable with the collection of shreds of evidence; or when logical reasoning in science is generally accepted by a scientific community. The other small group, in a similar notion, stated that the accumulation from everyday experiences, knowledge, and facts strengthen the stability of scientific knowledge. Contrary to this stand, merely 2.2% of them disagreed with the statement that “accumulation of evidence makes scientific knowledge more stable”. For them, no matter what, the accumulation of shreds of evidence has no guarantee in making scientific knowledge strong and absolute. As per one of the Bhutanese science teachers:

*“Scientific discovery and inventions happen at any time. The more research is carried out, the more knowledge comes out now and then. This adds to the existing body of knowledge and makes science a body of an ever-increasing amount of knowledge. For example, astronomy is one area where many discover of stars, galaxies, and planetary bodies happen now and then”.*

Many (71.6%) Bhutanese science teachers held uninformed views of the scientific model. Out of their mixed and confused conceptions, they pointed out that scientific models, irrespective of types, are exact copies of realities. With such a claim, they had little or no understanding that scientific models, either wholly or in part, are products of scientists’ creativity or imagination. Therefore, with a distorted view, a large majority of them believed that scientific models, in any case, are developed only with hard fact pieces of evidence or after conducting rigorous research.

#### Perceptions of the NOS: scientific method

Bhutanese science teachers’ perceptions of the NOS concerning the scientific method are presented in Table 4.

**Table 4:** Perceptions of the NOS: Scientific Method (*N* = 225)

Items	Response (%)		
	Agree	Uncertain	Disagree
Item 5: The scientific method is a fixed step-by-step process	49.5	15.2	35.3
Item 6: Science and the scientific method can answer all questions	19.3	26.9	53.8
Item 7: Scientific knowledge comes from experiments only	39.1	10.2	50.7

Nearly one-half (49.5%) of the Bhutanese science teachers held naive ideas of the scientific method. With their steadfast belief, they maintained that the scientific method, unlike other aspects of science, is a prescribed fixed step-wise method followed by scientists to attain quality results. With such a notion, they were of the view that scientists and the scientific community would largely fail to achieve effective results with creativity and imagination. On the other hand, little more than one-half (53.8%) of the Bhutanese science teachers disagreed with the idea that “science and scientific method can answer all questions”. They claimed that there is no plausible or intelligible explanation for many phenomena, such as the mystery of the Bermuda Triangle or why one often dreams while asleep. However, nearly two-fifths (19.3%) of the Bhutanese science teachers still believed that science can answer all questions. Indeed, they seemed quite certain when they voiced out that it takes time to understand certain phenomena before scientists come up with reliable answers. One of the Bhutanese science teachers commented about the scientific method as:

*“One should not do science from every possible angle. Experiments in science must be based on certain rules or orders. When one has to conduct experiments, one needs to make clear aims, procedures, and result recording sheets or tables. One cannot expect to have a better result if one or a few steps are missed out or not in proper order”.*

More than one-half (50.7%) of the Bhutanese science teachers held a contemporary view regarding the source of scientific knowledge. As indicated by them, scientific knowledge, both by and of itself, comes from many sources. As this was the case, they felt that scientific knowledge comes not only from experiments but also from other sources, such as educated guesses, imagination, and creativity. As opposed to this claim, however, 39.1% of Bhutanese science teachers expressed misinformed views. In their view, experiments are the only source of scientific knowledge. With this perception, they felt that scientists often develop hypotheses about the observed phenomena and carry out investigations to either confirm or disprove them. Moreover, they also pointed out that observations and experimentations are the two valid sources of scientific knowledge.

#### Perceptions of the NOS: Scientist’s work

Bhutanese science teachers’ perceptions of the NOS concerning scientists’ work are presented in Table 5.

**Table 5:** Perceptions of the NOS: Scientist’s Work (*N* = 225)

Items	Response (%)		
	Agree	Uncertain	Disagree
Item 10: Scientists do not use creativity and imagination in developing scientific knowledge	6.7	13.3	80
Item 11: Scientists are open-minded without any biases	42.7	28	29.3

Many (80%) Bhutanese science teachers expressed informed views about scientists’ works. As their understanding was quite coherent and sophisticated, they explained that scientific knowledge is a product of human endeavour. According to them, scientific knowledge, though largely empirical (e.g., observations and investigations), comes often from one’s imagination, educated guess; or a great deal of creative thinking and innovation by scientists. One of the Bhutanese science teachers maintained that:

“*Science is often based on pieces of evidence. However, observations or experiments are neither absolute nor final basis of science. Many scientific concepts, ideas, and beliefs are indeed based on certain elements of imagination and creativity*. *One very good example of this aspect is the idea regarding light reactions and dark reactions of photosynthesis*. *Also in physics, concepts related to black holes or twin paradox are all created based on scientists’ imagination”.*

Quite many (42.7%) Bhutanese science teachers held the misattributed view that “scientists are open-minded without any biases”. They thought that scientists, by far any profession, are not only rational but also objective in any sphere of works. In this connection, many of them remarked that science is bound to fail to obtain valid or accurate results if scientists are not open-minded or free of biases. Therefore, to most of them, scientists are never influenced by personal matters, such as ethnicity, religion, or societal values. However, nearly one-third (29.3%) of the Bhutanese science teachers held the correct view that scientists are neither free of biases nor completely open-minded.

#### Perceptions of the NOS: scientific enterprise

Bhutanese science teachers’ perceptions of the NOS concerning scientific enterprise are shown in Table 6.

**Table 6:** Perceptions of the NOS: Scientific Enterprise (*N* = 225)

Items	Response (%)		
	Agree	Uncertain	Disagree
Item 12: Science and technology are identical	55.5	13.5	31
Item 13: Scientific enterprise is an individual enterprise	18.2	29.8	52
Item 14: Society, politics, and culture do not affect the development of scientific knowledge	18.2	10.7	71.1

Many Bhutanese science teachers held incorrect views of interactions between science and technology. For instance, little more than one-half (55.5%) of them believed that science and technology, as these terms imply, are identical to each other. In their argument, most of them stated that technology is either the product of science or an applied part of science. In one of the instances, many of them pointed out that there would be virtually no marvels in technology, such as mobile phones and televisions, without the development and growth in scientific fields. The contemporary view that “science and technology are not identical” was expressed by just more than one-third (31%) of the Bhutanese science teachers.

Slightly more than one-half (52%) of the Bhutanese science teachers disagreed with the statement that “scientific enterprise is an individual enterprise”. As was the case, their expression was from the view that any progression in the scientific endeavour is always the product of a joint and collaborative venture. Interestingly, 71.1% of them disagreed with the statement that “society, politics, and culture do not affect the development of scientific knowledge”. In their view, the vast majority of them, in fact, expressed that scientific knowledge is considerably influenced by societal elements, such as social, political, and cultural aspects of society. As a supplementary note, they maintained that societal elements, to a greater extent, either support or impede the development of science. As evident in their justification, most of them actually felt that scientific advancement happens best when power structures within the government remain open to any innovation. The excerpt given below is the response of one of the teachers concerning the role of religion, politics, and culture:

*“Science is very much linked with societal factors. For example, religion is one thing that interferes with science. If one sect of the religious community feels as opposed to science, the community would either try to block or clamp down the idea of science. This goes well with politics and other cultural elements too”.*

### Perceptions based on compared means

#### Independent sample t-test

The comparison of Bhutanese science teachers’ perceptions of the NOS based on gender is shown in Table 7, while the statistical significance of their compared means is shown in Table 8.

**Table 7:** Mean Score of Bhutanese Science Teachers’ Perceptions of the NOS Based on Gender

Gender	N	Mean	Std. Deviation	Std. Error Mean
Score Male	157	15.68	4.51	.36
Female	68	16.12	5.28	.64

**Table 8:** Independent Samples t-Test on Bhutanese Science Teachers’ Perceptions of the NOS

		Levene’s Test for Equality of Variances		t-test for Equality of Means						
		F	Sig.	t	df	Sig. (2-tailed)	Mean Difference	Std. Error Difference	95% Confidence Interval of the Difference	
									Lower	Upper
Score	Equal variances assumed	.224	.637	−.632	223	.528	−.436	.690	−1.796	.923
	Equal variances not assumed			−.593	111.118	.554	−.436	.7345	−1.892	1.020

As shown in Table 7, the mean (M) score of Bhutanese male science teachers’ perceptions of the NOS was 15.68 (SD = 4.51), while the mean (M) score of Bhutanese female science teachers’ perceptions of the NOS was 16.12 (SD = 5.28).

The statistical significance of the compared mean scores between Bhutanese and female science teachers’ perceptions of the NOS is shown in Table 8. As per the Independent Sample *t*-test, there was no statistically significant difference in the perceptions of the NOS for Bhutanese female (M = 16.12, SD = 5.28) and male (M = 15.68, SD = 4.51) science teachers; t (223) = −.632, *p* = .528, 95% confidence interval (− 1.796 to .923).

#### One-way ANOVA test

The comparisons between Bhutanese science teachers’ perceptions of the NOS based on academic qualifications are shown as:

As shown in Table 9, Bhutanese science teachers’ perceptions of the NOS as per their academic qualifications appeared different from each other. The mean (M) score of Bhutanese science teachers with B. Ed qualification was 15.53 (SD = 4.427), PGDE with 16.72 (SD = 4.731), master with 16.03 (SD = 4.666), and PhD with 12.00 (SD = 7.036). The differences between the mean scores of NOS perceptions held by Bhutanese science teachers with B. Ed, PGDE, master, or PhD were statistically significant as determined by the One-way ANOVA (F (3, 221) =2.730, *p* = .045 (Table 10).

**Table 9:** Mean Score of Bhutanese Science Teachers’ Perceptions of the NOS Based on Qualification

	N	Mean	Std. Deviation	Std. Error	95% Confidence Interval for Mean		Minimum	Maximum
					Lower Bound	Upper Bound		
B.Ed	90	15.53	4.427	.467	14.61	16.46	2	26
PGDE	47	16.72	4.731	.690	15.33	18.11	8	36
Master	79	16.03	4.666	.525	14.98	17.07	5	26
PhD	9	12.00	7.036	2.345	6.59	17.41	3	22
Total	225	15.81	4.747	.316	15.19	16.44	2	36

**Table 10:** One-way ANOVA on Bhutanese Science Teachers’ Perceptions of the NOS

	Sum of Squares	df	Mean Square	F	Sig.
Between Groups	180.406	3	60.135	2.730	.045
Within Groups	4867.754	221	22.026		
Total	5048.160	224			

The Post-hoc comparison using the Tukey HSD test (Table 11) compared all pairs of the mean scores and determined if they were significantly different from the other. As indicated by the Tukey Post-hoc test, the differences between the paired mean scores of Bhutanese science teachers’ perceptions of the NOS were statistically not significant. However, the test revealed that the difference between the paired mean scores of Bhutanese science teachers with PGDE (M = 16.72, SD = 4.731) and PhD (M = 12.00, SD = 7.036) was statistically significant with *p <* .05*.*

**Table 11:** Tukey HSD Post-hoc Test on Bhutanese Science Teachers’ Perceptions of the NOS

(I) Qualification	(J) Qualification	Mean Difference (I-J)	Std. Error	Sig.	95% Confidence Interval	
					Lower Bound	Upper Bound
B.Ed	PGDE	−1.190	.845	.495	−3.38	1.00
	Master	−.492	.724	.905	−2.37	1.38
	PhD	3.533	1.641	.140	−.71	7.78
PGDE	B.Ed	1.190	.845	.495	−1.00	3.38
	Master	.698	.865	.851	−1.54	2.94
	PhD	4.723^*^	1.708	.031	.30	9.14
Master	B.Ed	.492	.724	.905	−1.38	2.37
	PGDE	−.698	.865	.851	−2.94	1.54
	PhD	4.025	1.651	.073	−.25	8.30
PhD	B.Ed	−3.533	1.641	.140	−7.78	.71
	PGDE	−4.723^*^	1.708	.031	−9.14	−.30
	Master	−4.025	1.651	.073	−8.30	.25

## Discussion

Findings from this study are discussed and interpreted as:

### Perceptions based on the measure of frequency

#### Perceptions of the NOS: scientific knowledge

Many Bhutanese science teachers believed that hypotheses are often, if not, always developed to become theories. While this was their view, they also assumed that theories, in any event, are developed with the hope to become laws. These, according to them, happen with the availability of the evidence or advancement in an understanding. Additionally, a substantial number of them also believed that scientific laws, by far, hold a more secured position than scientific theories. In general, these findings indicate that Bhutanese science teachers had not only a simplistic understanding but also a hierarchical view of the relationship amongst hypotheses, theories, and laws. These findings, by nature, are consistent with reports from a prior study that examined Bhutanese science teachers’ views of the NOS (Wangdi et al., 2019). In studies conducted by Aslan and Tasar (2013) and Dogan and Abd-El-Khalick (2008), a large majority of Turkish science teachers expressed a hierarchical view of the relationship amongst hypotheses, theories, and laws. In general, the notions that hypotheses become theories and theories into laws or “laws are matured theories fables” are certainly inappropriate and incoherent. This is because, amongst other things, theories and laws in science are different kinds of knowledge, serve different purposes, and one cannot be developed and transformed into the other. Moreover, hypotheses, theories, and laws neither share hierarchical relationships nor does one occupy higher status than the other. As is the case, “scientists do not usually formulate theories in the hope that one day they will acquire the status of law” (Lederman et al., 2013, p. 140). Typically, both theories and laws explain about the observed phenomena, but theories do not become laws (National Research Council, 2013).

Bhutanese science teachers in this study viewed “scientific knowledge is permanently subject to change” (Kremer et al., 2014, p. 2) or “scientific knowledge is tentative” (Capps & Crawford, 2013, p. 1950). Their justification was from the point that with new evidence or more advanced understanding, the established body of knowledge, such as theories, laws, or principles becomes the subject of modifications, revision, or even to the extent of rejection. As a matter of fact, Bhutanese science teachers’ understanding, at least to this extent, is true. The advancement of scientific knowledge, including theories and laws, relies heavily on repeated facts, observations, or the collection of pieces of evidence. Moreover, scientific knowledge is open to change or revision as in light of new evidence, advances in theory or old evidence are interpreted again in light of new theoretical advances (Abd-El-Khalick, 2012; Bell, 2009, National Science Teaching Association, 2013; Wahbeh & Abd-El-Khalick (2014). Therefore, scientific knowledge, though durable and reliable, is never absolute and certain. In recent studies conducted by Torres et al. (2015) and Torres and Vasconcelos (2015), Portuguese pre-service and in-service science teachers also expressed similar views. Like Portuguese science teachers, Bhutanese science teachers in this study expressed scientific knowledge as “never absolute or certain”. Portuguese science teachers, however, have admitted further that scientific knowledge is quite “durable and reliable” (Abd-El-Khalick, 2012, p. 375).

Contrary to a common belief, one must also realise that not all scientific knowledge is subject to change. Perhaps, the claim that scientific knowledge is tentative may wrongly lead to an understanding that all scientific knowledge is temporary or non-durable (Hodson & Wong, 2017). This issue was raised recently in the report of Nature (2017) that “the way the NOS is taught in schools is that it encourages rather too much doubt over scientific ideas. Not all science is tentative … (p. 149). Conversely, this study lacks evidence to substantiate if Bhutanese science teachers also held the view that “not all science is tentative” (p. 149). As is the case, such views are also rarely reported in international literature.

Conditionally, many Bhutanese science teachers largely believed in the “Baconian induction” of science (McComas et al., 1998, p. 58). As this was their notion, they seemed deeply entrenched with their steadfast belief that “accumulation of evidence makes scientific knowledge more stable”. While justifying their claim, they voiced out two lines of thought. A sizable number of them opined that scientific knowledge becomes more stable when there is enough evidence gathered or when logical reasoning in science itself is generally accepted by the scientific community. This claim by Bhutanese science teachers appears quite appropriate and sophisticated. At the core, one of the characteristics of science is its cumulative nature. As more research and discoveries are made, “we progressively come to a more and more complete understanding of the physical universe” (Zeigler, 2012, p. 585). These findings mirror Akerson and Donnelly’s (2008) reports on US pre-service science teachers’ beliefs of the NOS. However, what is interesting is that science teachers of this study and those of Akerson and Donnelly (2008) did not realise that there is no guarantee in the “Baconian induction” of science. As opposed to a common belief, the developmental pathway of scientific knowledge is such that it can often be accretionary, revisionary, or even goes through jumps (Brickhouse, 1990; Duschl & Grandy, 2013; Haidar, 1999). Therefore, “knowledge of science, no matter how much supporting evidence exists, may change in the future for these reasons” (Lederman, 2007, p. 835).

On the other hand, it could be seen that the overwhelming majority of Bhutanese science teachers held naive views regarding the stability of scientific knowledge. Similar to the finding of the Ma (2009) in Chinese secondary science teachers, the considerable number of Bhutanese science teachers in this study also largely believed that the daily accumulations of experiences, facts, or knowledge improve the stability of scientific knowledge. For them, individual pieces of information or facts are gathered and examined until a theory or a law is discovered. These views by them seem quite naive in that knowledge and facts cannot be assumed as valid as evidence. Therefore, unless backed by evidence or accepted by the scientific community, scientific knowledge built out of human creativity or imagination may typically increase the amount of knowledge but not necessarily augment stability and credibility.

Significantly, many Bhutanese science teachers believed that scientific models are exact copies of realities. As their views were mostly mixed and confused, for them, scientific models are principally, but exclusively never created out of imagination, creativity, and educated guesses. With such views, they ascribed the notion that if scientific models are not exact replicas of realities, science would neither progress further nor can inform the world with truth. For instance, they described how Bohr’s atomic model typically depicts the arrangement of electrons, protons, and neutrons in atoms. This group of Bhutanese science teachers, thus, lacked the idea that many scientific models, irrespective of types and uses, are legitimate products of a scientist’s creativity, imagination, educated guesses. With theoretical functional purpose, scientific models, to certain extent, represent realities that remain virtually no direct experience with our sensory apparatus. By the same token, many scientific models serve as analogs in explaining concepts that are increasingly counterintuitive and incomprehensible. As opposed to being exact replicas of realities, scientific models, thus, take a great deal of human invention or “partly the product of inference, imagination, and creativity” (Sumranwanich & Yuenyong, 2014, p. 2444). For example, “scientific concepts, such as atoms, black holes, and species, are functional theoretical models rather than faithful copies of realities” (N. Lederman (2007, p. 834). In Turkey, Mihladiz and Dogan (2014) ascertained elementary science teachers subscribing to similar incorrect views. These findings also mirror reports from past research conducted by Torres et al. (2015) and Torres and Vasconcelos (2015) in Portugal. As indicated by their studies, science teachers in Portugal, however, believed that the construction of models happens only after conducting rigorous reviews or experimentation. This particular view, however, did not come out explicitly from the findings of this study.

#### Perceptions of the NOS: scientific method

The considerable number of Bhutanese science teachers had the realisation that science is a universal step-by-step process. Their argument was fueled, in part, by the belief that science would certainly fail to come up with quality results if scientists use creativity or imagination. As per Bell et al. (2016), there is no single sequence of practical, conceptual, or logical activities that will unerringly lead to valid claims, let alone certain knowledge. Findings from this study, therefore, demonstrate that many Bhutanese science teachers have failed to realise that “there is no single scientific method that would guarantee the development of infallible knowledge” (Abd-El-Khalick, 2012, pp. 357–358). In the United States of America (USA), a recent study conducted by Bell et al. (2016) observed pre-service master teachers struggling with similar misconceptions. Also, many in-service science teachers in the USA (Akerson & Donnelly, 2008; Capps & Crawford, 2013), Turkey (Aslan & Tasar, 2013), and Palestine (Wahbeh & Abd-El-Khalick, 2014) were reportedly found with such incorrect views. In contrast, Torres and Vasconcelos (2020), however, observed many Portuguese pre-service science teachers with informed views of the scientific method.

In science education literature, there is a general belief that science teachers’ notion of “scientists follow a single method” is perpetuated mostly by prescribed science textbooks (Wahbeh & Abd-El-Khalick, 2014), confirmatory or structured laboratory activities (Capps & Crawford, 2013), or continued and explicit emphasis that assume universal methods (Abd-El-Khalick et al., 2008). These theoretical assumptions of science education literature, as they imply, may equally hold with the case of Bhutanese science teachers’ notion. Therefore, typically, but not necessarily, one can assume that Bhutanese science teachers’ notion of “scientists follow a single method” is perpetuated by prescribed curricular designs and science learning activities that promote prescribed methods of inquiry. Recent studies conducted by Dorji et al. (2020) and Dorji (2015) found explicit use of prescribed curricular designs with structured learning cycles in many parts of Bhutanese schools.

As found out by Bartos and Lederman (2014), it is quite noteworthy that the substantial number of Bhutanese science teachers disagreed with the statement that “science and the scientific method can answer all questions”. Their understanding was based largely on the fact that science, in many ways, lacks tenable explanations for many natural events, such as dreams and the Bermuda triangle. Intriguingly, while they agreed that “science cannot answer all questions” (National Research Council 2013, p. 6), they believed that scientists can still come up with explanations at one point in time. A study conducted by Ma (2009) also found Chinese secondary school science teachers with a similar notion. Overall, views expressed by Bhutanese science teachers appeared certainly true, as it is an obvious fact that “science cannot answer all the questions” (National Research Council 2013, p. 6). Conversely, quite a large number of Bhutanese science teachers still believed that science has explanations for every question. Ordinarily, but not necessarily, such a notion by them may be ascribed to the stereotype prevalent in the Bhutanese educational settings. For instance, it is quite common in Bhutan that “any discoveries or scientific breakthroughs are highly interlinked with no other subject but science” (Wangdi et al., 2019, p. 87).

As observed by Bell et al. (2011) in US pre-service science teachers, many Bhutanese science teachers held contemporary views about the source of scientific knowledge. As their understanding was quite matured, scientific knowldge by no means solely comes from experiments exclusively. To many of them, a bulk of scientific knowldge, in many instances, is constructed out of one’s educated guesses, imagination, and creativity. As is the case, while “scientific knowledge is, at least partially, based on and/or derived from observations of the natural world (i.e., empirical)”, it nevertheless involves a great deal of human imagination and creativity (Lederman, Antink, and Bartos, 2014, p. 288). Therefore, science as opposed to a common belief is not completely lifeless, rational, and orderly prescribed activity. Science involves the invention of explanations and the generation of ideas and this requires a great deal of human endavour. On the other hand, there was another group of Bhutanese science teachers who still thought that “science is empirical” (Lederman et al., 2002, p. 500). Their notion was propelled, in part, by the belief that the development of scientific knowledge involves making empirical studies, such as observations and investigations. These findings are consistent with a prior study that examined Palestinian science teachers’ understanding of the NOS (Wahbeh & Abd-El-Khalick, 2014). In contrast to this common notion, many scientists, on most occasions, lack direct access to many natural phenomena. In such cases, scientists’ observations are often filtered through their human perceptual apparatus, mediated by assumptions underlying the functioning of scientific instruments, and/or constructed from within theoretical frameworks.

#### Perceptions of the NOS: scientists’ work

As opposed to an understanding of science teachers in the USA (e.g., Akerson and Donnelly, 2008; Akerson et al., 2006), quite a large number of Bhutanese science teachers in this study accepted creativity and imagination as part and parcel of scientists’ work. This supports their earlier claim that generating scientific knowledge involves a great deal of human imagination and creativity.

Consistent with Thai pre-service and in-service science teachers’ (Buaraphan, 2009; Prachagool & Nuangchalerm, 2019) and Portuguese science teachers’ views (Torres & Vasconcelos, 2019) Bhutanese science teachers felt that scientists are largely open-minded and free of biases. With diehard myth, a sizable number of them argued that science would fail in many areas of inquiry to attain quality inferences if scientists are not free of personal assumptions or biases. This incorrect view of Bhutanese science teachers, thus, attest to their deep entrenchment with the myth that “scientists are objective in their work”. According to Abd-El-Khalick, (2012, 2014), scientists as human beings, like all of us, are greatly influenced by their intrinsic commitments, beliefs, personal experiences; or expectations. Therefore, oftentimes, scientists’ choice of problems to investigate and methods of investigations, observations, and interpretations are deeply affected by their backgrounds.

#### Perceptions of the NOS: scientific Enterprise

The sizable number of Bhutanese science teachers held the misinformed view that “science and technology are identical”. As they were shrouded with the steadfast belief, they assumed that science and technology are the same entity; technology is a legitimate product of science, or technology is a mere type of applied science. These claims by Bhutanese science teachers are consistent with the prior research that examined Thai (Buaraphan, 2010; Promkatkeaw et al., 2007) and US (Herman et al., 2017) science teachers’ conceptions of the NOS. At the core, such types of notions are widely believed to be offshoots of strong cultural roots. In a usual setting, people often infer from artefacts and systems that trail scientific breakthroughs. Take, for example, “atomic physics leading to the development of nuclear power” (Buaraphan, 2009, p. 208) is what many people think. In an actual sense, progress in any field of scientific endeavour is the result of a seamless combination of science, mathematics, and technology (American Association for the Advancement of Science, 2010). Therefore, science, mathematics, and technology have characters and histories of their own, where each one is independent but complementary to the other.

As opposed to reports of Wangdi et al. (2019), quite many Bhutane science teachers in this study had disagreed that “scientific enterprise is an individual enterprise”. As per their understanding, science, like any other discipline, is a perfect niche of joint ventures, where progress in it is the legitimate product of collaborative efforts. A few of them, with matured understanding, further admitted that an individual’s pursuit, while worthy, is often met with increasing challenges. Theoretically, most concepts in science are inherently complex to be pursued alone. Perhaps, endavour by scientists require institutional checking by scientific communities before considering valid or credible scientific knowledge (Kartal et al., 2018). Scientific ideas that arise in the minds of individual scientists are, thus, first screened out by individuals themselves before being projected to be considered by the scientific community (Lederman and Lederman, 2014).

Science as a human endeavour is largely shaped by various societal values (Torres & Vasconcelos, 2015, 2016). Similar to this, the large number of Bhutanese science teachers expressed that “scientific knowledge is socially negotiated” (Abd-El-Khalick, 2012, p. 358). Science, as indicated by their justification, is purely a human construct developed in the context of a larger cultural setting. Scientists, according to this group of Bhutanese science teachers, are products of either social or cultural values or both. These views by Bhutanese science teachers correspond to the postulation of Bell et al. (2012) about scientific knowledge. According to Bell et al. (2012), scientific knowledge is developed in the context of society and culture; and ways of funding, technological innovations, and societal problems fuel the need for certain investigations. A study conducted by Capps and Crawford (2013) also observed similar conceptions held by science teachers in the USA. Indeed, science as a human construct follows, affects, and is affected by various elements in which it is embedded. These elements include, but are not limited to social values, power structures, politics, socio-economic factors, philosophy, and religion (Akerson et al., 2006). Government research grants, by and large, are believed to bring positive influence in advancing scientific knowledge. As opposed to this, cultural and religious values, to certain extent, are supposed as social factors that impede scientific progression (Pavez et al., 2016).

### Perceptions based on compared means

The mean score of Bhutanese female science teachers’ perceptions of the NOS (M = 16.12, SD = 5.28) was higher than the mean score of Bhutanese male science teachers’ perceptions of the NOS (M = 15.68, SD = 4.51). However, the Independent Sample *t*-test showed that there is no statistically significant difference in the perceptions of the NOS between Bhutanese males (M = 15.68, SD = 4.51) and females (M = 16.12, SD = 5.28) science teachers; t (223) = −.632, *p* = .528. These findings suggest that Bhutanese science teachers’ perceptions of the NOS do not depend upon their gender. A similar finding was also observed by Amer Saif (2016) in Saudi Arabia, Oluwatayo (2011) and Taale (2014) in Nigeria, and Yaman and Nuhoglu (2010) in Turkish contexts.

In theory, male and female science teachers embedded in a similar socio-cultural setting are invariably assumed to possess similar understanding of the NOS (Dogan & Abd-El-Khalick, 2008; Kang et al., 2005). This theoretical assumption may significantly hold true with similar views of the NOS shared by Bhutanese male and female science teachers. This is because all the Bhutanese science teachers who took part in this study came straight away from similar Bhutanese cultural settings. Therefore, Bhutanese male and female science teachers’ similar views of the NOS can assumedly be ascribed as the sheer sum result of their similar cultural background. Categorically, there is also a standing belief that teachers trained through the same curricula, same educator, or processes markedly possess similar views of the NOS (Adedoyin & Bello, 2017; Ajaja, 2012; Saif, 2016). Similar to these propositions, most Bhutanese science teachers, both in schools and colleges, receive training through the same curricula. Categorically, it appears quite certain that similar views of the NOS shared by Bhutanese science teachers irrespective of their gender are some typical results of the same curricula offered in schools and colleges.

Concurrently, the levels of Bhutanese science teachers’ perceptions of the NOS based on academic qualifications appeared different from each other. The mean (M) score of science teachers with B. Ed qualification was 15.53 (SD = 4.427), PGDE with 16.72 (SD = 4.731), master with 16.03 (SD = 4.666), and PhD with 12.00 (SD = 7.036). As indicated by the One-way ANOVA test (F (3, 221) =2.730, *p*=. 045, differences between these mean scores were statistically significant. Therefore, these findings imply that the levels of NOS perceptions held by Bhutanese science teachers holding B. Ed, PGDE, master, or PhD qualifications are significantly different from one to another. With such a result, one may presumably build an impression that the levels of NOS perceptions are typically influenced by academic qualifications. Similarly, a study by Waters-Adams (2006) observed differing levels of NOS perceptions held by United Kingdom (UK) science teachers with different academic qualifications. However, as his study was purely qualitative, there is no clue of any further inquiry from a statistical point of view.

Conversely, as revealed by the Tukey Post-hoc test, differences between the paired mean scores of Bhutanese science teachers holding different academic qualifications were statistically not significant. The only statistically significant difference was observed between the paired mean scores of Bhutanese science teachers holding PGDE (M = 16.72, SD = 4.731) and PhD (M = 12.00, SD = 7.036) with *p <* .05. This finding, as it implies, indicates that Bhutanese science teachers holding PGDE had better conceptions of the NOS than those with PhD. In Turkey, Dogan and Abd-El-Khalick, 2008 observed Turkish science teachers with PhD with the most misinformed views of the NOS followed by teachers with master’s and bachelor’s degrees. However, their studies have chosen to report exclusively from a qualitative point of view.

By and large, there is no significant relationship between science teachers’ conception of the NOS and their corresponding academic qualifications (Carey & Stauss, 1970; Lederman, 1992; Mellado, 1997). Science teachers’ conceptions of the NOS are, rather, believed to be dependent on the amount of NOS learning opportunities received by them in their teacher preparation programmes (Ajaja, 2012; Buckermann et al., 2018). This assumption stems from the view that if science teachers with bachelor’s degrees receive more exposure to the NOS, they are assumed to have better understanding of the NOS than those with master’s degrees or PhD (Ajaja, 2012). Typically, but not necessarily, this theoretical stand might serve as one possible reason ascribing why Bhutanese science teachers with PGDE had better understanding of the NOS than those with PhD.

## Conclusions

Bhutanese science teachers’ conceptions of the NOS appeared incoherent and unsophisticated. As their views were mostly shallow, many of them thought that hypotheses are often developed with a hope to become scientific theories. By the same token, for quite many of them, scientific theories, in any event, are always converted into laws. They also held the uniformed view that laws occupy much more secured position than scientific theories. Meanwhile, they also felt that accumulations of daily experiences, facts, and knowledge increase the stability of scientific knowledge. More so, as their knowledge on the scientific model was uncertain, many of them expressed scientific models as exact replicas of natural phenomena.

Bhutanese science teachers, with their diehard myth, felt that the scientific method is a single, universally fixed, and a step-by-step process. While they thought of experiments as the absolute and ultimate source of scientific knowledge, science and scientists, as per them, are irrefutably objective and open-minded. Moreover, they also held an immature view, whereby they firmly believed science and technology as a single entity.

The Independent Sample *t*-test showed that there is no statistically significant difference between the levels of NOS perceptions held by Bhutanese male and female science teachers with *p* > .05. The One-way ANOVA test showed statistically significant differences amongst the levels of NOS perceptions held by Bhutanese science teachers with different academic qualifications. However, as determined by the Tukey Post-hoc test, the only statistically significant difference was observed between the paired mean scores of Bhutanese science teachers holding PGDE and PhD with *p <* .05.

### Limitations

Although this study collected data from 225 Bhutanese science teachers from four major regions of Bhutan, findings in themselves may not represent the view of the whole Bhutanese science teacher population. This is because this study could not draw representative samples based on the probabilistic sampling design. Moreover, this study had some coverage errors, wherein many representative samples could not be drawn into the study due to the lack of a multimode design.

### Educational implications

Findings from this study suggest that the science curriculum that requires science teachers to have an in-depth understanding of the NOS is one of the ways to keep science teachers well informed about the NOS. Indeed, science teachers cannot help students understand what they do not understand. There is a widespread recognition that science teachers’ understanding of the NOS determines how they influence their students’ conceptions of the NOS (Lederman & Lederman, 2019a).

The science curriculum that engages teachers in authentic inquiry-based approaches is believed to promote science teachers’ conceptions of the NOS. Indeed, there is a widespread recognition that the NOS is best taught within a context of scientific inquiry or activities that are reasonable facsimiles of inquiry (Lederman, 2007). However, there is also a general understanding that participating in scientific inquiry does not implicitly teach either teachers or students about the NOS. Therefore, the science curriculum may advocate the idea of intentionally planning to teach and assess tangible aspects of the NOS content rather than just engaging in doing science or in episodes of the history of science (Abd-El-Khalick & Lederman, 2000).

Findings from this study also have some important implications for teacher professional development (PD) programmes. A well-crafted PD programme that engages teachers in authentic opportunities in planning lesson plans on the aspects of the NOS may be one of the viable approaches. Therefore, it is always better if teachers are provided several opportunities to make plans and implement lessons on the aspects of the NOS. There is a general understanding that PD programmes that focus on microteaching sessions not only improve teachers’ content knowledge but also pedagogical knowledge of the NOS (Capps & Crawford, 2013).

Lastly, our findings also have important suggestions for pre-service teacher preparation courses. The teacher training modules, particularly at colleges of education, may also choose to expose prospective science teachers to both contents and processes of integrating the NOS in the classroom teaching-learning process. This may be achieved through designing teacher preparation course contents that advocate the idea of intentionally planning to teach and assess tangible aspects of the NOS content and the process of doing science. Indeed, there can be wide arrays of teacher preparation course contents that enable prospective science teachers to immerse in microteaching sessions to improve both content knowledge and pedagogical knowledge of the NOS.

Overall, the Bhutanese science curriculum, professional development programmes, and teacher preparation course contents are crafted to help science teachers understand the following aspects of the NOS:

1. Hypotheses, theories, and laws serve different purposes in science. They do not share a hierarchical relationship, whereby hypotheses are never developed to become theories and laws. Laws are neither glorified theories nor matured theories fables. Therefore, laws do not hold higher status than theories and vice-versa.
2. Scientific knowledge is tentative to change. However, not all scientific knowledge is subject to change. Therefore, there is certain scientific knowledge that is neither absolutely temporary nor non-durable.
3. The accumulation of evidence makes scientific knowledge more stable. However, there is no guarantee that the accretion of evidence makes scientific knowledge always stable. The developmental nature of scientific knowledge is rather revisionary or even leapfrogs from one form to another.
4. The accumulation of daily experiences, facts, or knowledge increases or adds upon the pre-existing knowledge of science. However, this does not necessarily enhance the stability of scientific knowledge. Indeed, daily experiences, facts, or knowledge are not as valid or credible as evidence.
5. Scientific models are neither identical copies nor replicas of realities. Many scientific models are rather products of a scientist’s imagination or educated guesses; built to explain realities rather than complete replicas of observed phenomena.
6. There is no single, fixed step-by-step process, universal, or recipe-like process of doing science. Scientists use varieties of methods to construct scientific knowledge.
7. Science is neither absolute nor certain. Science, in many ways, lacks plausible or tenable explanations for many natural events. Therefore, science cannot answer all questions.
8. Scientific knowledge is not just empirical. Perhaps, it involves a great deal of human imagination and creativity. Science, therefore, is not lifeless, rational, and orderly activities.
9. Scientists are neither free of biases nor immune to subjectivity. As scientific knowledge is largely theory-laden, the theoretical commitments, beliefs, previous knowledge, training, experiences, and expectations influence scientists’ work.
10. Science and technology are two different domains. Although science and technology complement each other, they are never the same entity or product of one another. Perhaps, they have a character and history of their own, where each one is independent of the other.

## Data Availability

All data generated or analysed during this study are included in this published article [and its supplementary information files].

## Abbreviations

NOS: Nature of science
BSc: Bachelor of science
NCE: Nigerian certificate of education
MOSQ: Myths of science questionnaire
ANOVA: One-way analysis of variance
NSTA: National Science Teaching Association
NRC: National Research Council
REC: Royal Education Council
B.Ed: Bachelor’s degree in education
PGDE: Post-graduate diploma in education
M.Ed: Master’s degrees in education
MSc: Master’s degrees science disciplines
PhD: Doctorate of philosophy
SPSS: Statistical Package for Social Sciences
USA: United States of America
AAAS: American Association for the Advancement of Science
M: Mean
PD: Professional development
